# Supplementary material for: Simultaneous evaluation of metabolomic and inflammatory biomarkers in children with different body mass index (BMI) and waist-to-height ratio (WHtR)
Source: PLoS One. 2020 Aug 24;15(8):e0237917. doi: 10.1371/journal.pone.0237917 (PMC7446833; doi:10.1371/journal.pone.0237917)
Supplement: S1 Table — Kruskal Wallis with Dunnett’s multiple comparison test were used to evaluate the parameters classified by BMI and, the U Mann Whitney test were used to evaluate parameters classified by WHtR. Significant differences (p < 0.05) between groups in both types of classification are shown with different letters. Gluc: Glucose, Ins: insulin, HOMA-IR: homeostasis model assessment insulin resistance, TG: triglycerides, TC: total cholesterol, LDL-C: low-density lipoprotein cholesterol, HDL-C: high density lipoprotein cholesterol, C0: carnitine, C2: acetyl carnitine, C4: isobutyril carnitine, C6: hexanoyl carnitine, C8: octanoyl carnitine, C10: decanoyl carnitine, C12: dodecanoyl carnitine, C14: tetradecanoyl carnitine, C16: palmitoyl carnitine, C18: octadecanoylarnitine (stearoylcarnitine), C3: propionyl carnitine, C5: isovaleryl carnitine, Gly: glycine, Ala: alanine, Met: methyonine, Leu: leucine, Val: Valine, Phe: phenylalanine, Tyr: tyrosine, Arg: arginine, Cit: citrulline, Orn: ornitine, Pro: proline. (DOCX) [file pone.0237917.s001.docx]

**S1 Table. Metabolic profile in schoolchildren classified by BMI or WHtR**

| **Biomarkers** | **BMI** | | | **WHtR** | |
| --- | --- | --- | --- | --- | --- |
|  | **NW** | **OV** | **OB** | **NG** | **CVR** |
| **Biochemical** | **Mean ± SD** | | | **Mean ± SD** | |
| **Gluc (mg/dL)** | 94 ± 9^a^ | 97 ± 9^a,b^ | 99 ± 6^b^ | 95 ± 9 | 98 ± 8^a^ |
| **Ins (µUI/mL)** | 1.9 ± 1.6 | 2.8 ± 2.0^a^ | 2.1 ± 1.6 | 1.9 ± 1.6 | 2.4 ± 1.8^a^ |
| **HOMA-IR** | 0.4 ± 0.4 | 0.7 ± 0.5^a^ | 0.5 ± 0.4 | 0.4 ± 0.4 | 0.6 ± 0.4^a^ |
| **TAG (mg/dL)** | 92 ± 41^a^ | 102 ± 43^a^ | 127 ± 49^b^ | 89 ± 40 | 120 ± 47^a^ |
| **TC (mg/dL)** | 140 ± 32^a^ | 144 ± 22^a,b^ | 155 ± 30^b^ | 140 ± 32 | 150 ± 27^a^ |
| **LDL-c (mg/dL)** | 65 ± 20^a^ | 70 ± 15^a,b^ | 78 ± 20^b^ | 64 ± 20 | 75 ± 19^a^ |
| **HDL-c (mg/dL)** | 47 ± 13 | 43 ± 12 | 43 ± 1 | 47 ± 14 | 42 ± 11^a^ |
| **Acylcarnitines** |  | | | | |
| **C0 (μmol/L)** | 15.5 ± 3.2 | 15.3 ± 3.8^a^ | 17.4 ± 3.6^b^ | 15.4 ± 3.2 | 16.5 ± 3.9 |
| **C2 (μmol/L)** | 0.75 ± 0.43 | 0.73 ± 0.52 | 0.71 ± 0.30 | 0.75 ± 0.42 | 0.72 ± 0.43 |
| **C4 (μmol/L)** | 0.05 ± 0.02 | 0.05 ± 0.02 | 0.06 ± 0.03 | 0.054 ± 0.02 | 0.06 ± 0.03 |
| **C6 (μmol/L)** | 0.02 ± 0.007 | 0.016 ± 0.007^a^ | 0.02 ± 0.006 | 0.019± 0.007 | 0.018 ± 0.007 |
| **C8 (μmol/L)** | 0.05 ± 0.06 | 0.04 ± 0.02 | 0.06 ± 0.08 | 0.05 ± 0.06 | 0.05 ± 0.06 |
| **C10 (μmol/L)** | 0.07 ± 0.05 | 0.06 ± 0.04 | 0.07 ± 0.05 | 0.07 ± 0.05 | 0.07 ± 0.05 |
| **C12 (μmol/L)** | 0.02 ± 0.008 | 0.02 ± 0.01 | 0.02 ± 0.008 | 0.021 ± 0.008 | 0.020 ± 0.009 |
| **C14 (μmol/L)** | 0.009 ± 0.003^a^ | 0.01 ± 0.002^a,b^ | 0.011 ± 0.003^b^ | 0.009 ± 0.003 | 0.011 ± 0.002^a^ |
| **C16 (μmol/L)** | 0.029 ± 0.009^a^ | 0.028 ± 0.01^a,b^ | 0.03 ± 0.01^b^ | 0.027 ± 0.009 | 0.03 ± 0.01^a^ |
| **C18 (μmol/L)** | 0.01 ± 0.00 | 0.01 ± 0.00 | 0.01 ± 0.00 | 0.01 ± 0.00 | 0.01 ± 0.00 |
| **C3 (μmol/L)** | 0.07 ± 0.03^a^ | 0.08 ± 0.03^a,b^ | 0.09 ± 0.03^b^ | 0.07± 0.03 | 0.08 ± 0.03^a^ |
| **C5 (μmol/L)** | 0.027 ± 0.01^a^ | 0.028 ± 0.01^a,b^ | 0.032 ± 0.01^b^ | 0.027 ± 0.01 | 0.029± 0.01 |
| **Amino acids** |  | | | | |
| **Gly (μmol/L)** | 187 ± 81^a^ | 163 ± 74^a,b^ | 163 ± 66^b^ | 179 ± 64 | 169 ± 86 |
| **Ala (μmol/L)** | 170 ± 56 | 176 ± 62 | 171 ± 50 | 168 ± 52 | 176 ± 60 |
| **Met (μmol/L)** | 13 ± 5 | 12 ± 5 | 14 ± 6 | 12 ± 4 | 14 ± 6 |
| **Leu (μmol/L)** | 82 ± 30 | 81 ± 35 | 95 ± 41 | 79 ± 28 | 91 ± 40 |
| **Val (μmol/L)** | 62 ± 20^a^ | 62 ± 20^a^ | 76 ± 25^b^ | 61 ± 17 | 71 ± 25^a^ |
| **Phe (μmol/L)** | 38 ± 18 | 35 ± 17 | 40 ± 22 | 36 ± 16 | 39 ± 22 |
| **Tyr (μmol/L)** | 40 ± 12^a^ | 41 ± 15^a,b^ | 49 ± 17^b^ | 40 ± 12 | 46 ± 16^a^ |
| **Arg (μmol/L)** | 11 ± 6 | 12 ± 7 | 16 ± 10 | 12 ± 7 | 14 ± 9 |
| **Cit (μmol/L)** | 10 ± 3 | 10 ± 4 | 10 ± 3 | 10 ± 3 | 10 ± 3 |
| **Orn (μmol/L)** | 53 ± 33 | 48 ± 30 | 76 ± 25 | 49 ± 23 | 54 ± 38 |
| **Pro (μmol/L)** | 92 ± 36 | 97 ± 45 | 98 ± 37 | 90 ± 29 | 100 ± 46 |
