# Supplementary material for: Simultaneous evaluation of metabolomic and inflammatory biomarkers in children with different body mass index (BMI) and waist-to-height ratio (WHtR)
Source: PLoS One. 2020 Aug 24;15(8):e0237917. doi: 10.1371/journal.pone.0237917 (PMC7446833; doi:10.1371/journal.pone.0237917)
Supplement: Attachment — Submitted filename: Response to reviewers.docx [file pone.0237917.s002.docx]

Mexico City, May 14,2020

**Rebuttal letter**

**Responses to Reviewer #1:**

Corrections were made and highlighted in green, as they appear in the file named “Revised manuscript with track changes”.

1. p-values with statistical significance were included in tables. We modified all Tables to clarify their content. Old table 4 is now Supplementary Table 1 (S1). New Table 4 describes multivariate analysis.
2. Two paragraphs were added in Discussion addressing Marginean's study (Lines 299-304).
3. All numbers in tables were converted to include a maximum of three significant digits.
4. Insulin values were added in New Table 4.
5. Number of measured amino acids was limited by the pre-designed commercial kit. However, we selected this kit because all groups of amino acids were represented according to their metabolic fate.
6. Bivariate analysis that included acylcarnitines concentrations (old Figure 4) was included in Supplementary Table 1

**Additional changes:**

Other corrections were made, they appear turquoise highlighted in the file named “Revised manuscript with track changes”.

1. Line 4: biomarkers instead of markers.
2. Line 8: corresponding author affiliation number.
3. Line 139: Gluc and TAG initials instead of Glucose and TG.
4. Line 149: initials of Insulin (Ins) was added.
5. Lines 151 and 152: initials of Gluc and Ins instead of the complete name.
6. Line 181, 192, 214, and 246: schoolchildren instead of school children.
7. Line 185, 196: initial definitions were included in the figure legend.
8. Line 189 -190, 250: NG initials instead of N.
9. Line 219- 227: all paragraph was modified.
10. Line 289 and 294: tyrosine and Val words were changed for phenylalanine, and ornithine such as valine (complete word).
